# Should dolutegravir always be withheld in people with HIV on dolutegravir with incident diabetes mellitus? a case report

**DOI:** 10.1186/s12879-023-08712-z

**Published:** 2023-10-30

**Authors:** Frank Mulindwa, Barbara Castelnuovo, Nele Brusselaers, Robert Bollinger, George Yendewa, Willington Amutuhaire, Claudine Mukashaka, Jean-Marc Schwarz

**Affiliations:** 1 Capacity Building Unit, Makerere University Infectious Diseases Institute, Kampala, Uganda; 2 Global Health Institute, Antwerp University, Antwerp, Belgium; 3 Centre for Translational Microbiome Research, Department of Microbiology, Tumour and Cell Biology, Karolinska University, Stockholm, Sweden; 4 School of Medicine, Johns Hopkins University, Baltimore, USA; 5 Department of Internal Medicine, Case Western Reserve University, Cleveland, USA; 6 School of Medicine, University of California San Francisco, San Francisco, USA; 7 Department of Basic Sciences, Touro University California College of Osteopathic Medicine, Vallejo, CA USA

**Keywords:** HIV, Integrase inhibitors, Dolutegravir, Accelerated hyperglycemia, Type 2 Diabetes Mellitus

## Abstract

Dolutegravir (DTG), an integrase strand transfer inhibitor is currently the recommended first and second line anti-retroviral therapy (ART) anchor agent by the World Health Organization due to its favorable side effect profile, high efficacy and genetic barrier to resistance.

Despite its very good side effect profile, there have been multiple case reports of ART experienced patients developing hyperglycemia within weeks to a few months after switching to DTG preceded by weight loss. At population level, however, DTG as well as other integrase inhibitors have been demonstrated to have a reduced risk of incident diabetes mellitus (T2DM) compared to other HIV drug classes.

Following multiple similar reports of accelerated hyperglycemia in Uganda during the first pilot year of DTG use, the Uganda Ministry of Health recommended withholding dolutegravir in all patients who develop diabetes. Whether this recommendation should be applied to all patients with incident T2DM remains to be demonstrated.

We present a clinical case of an HIV positive ART naïve man who was diagnosed with T2DM after 36 weeks on DTG. We describe changes in blood glucose, glycated hemoglobin, insulin resistance and pancreatic beta cell function before and after withholding DTG. We demonstrated that he was phenotypically different from the reported cases of accelerated hyperglycemia and he continued to have worsening insulin resistance despite withholding DTG. His blood glucose improved with dietary T2DM management. It is possible he had an inherent risk of developing T2DM independent of his exposure to DTG. This put in question whether DTG should universally be withheld in PLHIV with incident T2DM in Uganda.

## Background

Integrase strand transfer inhibitors (INSTIs) block integrase (an HIV enzyme) responsible for integration of viral DNA into the DNA of the host cell [1]. The World Health Organization (WHO) recommended the use of dolutegravir (DTG) an INSTI, as a preferred first and later second line anchor drug due to its high genetic barrier to resistance, efficacy and good side effect profile [2]. We demonstrated in a meta-analysis, reduced risk of incident type 2 diabetes mellitus (T2DM) in patients on INSTIs compared to those on non-nucleoside reverse transcriptase inhibitors (NNRTIs) and protease inhibitors (PIs) in different sub-populations apart from African populations who were largely underrepresented in the analysis [3]. These results were replicated in another meta-analysis [4]. In the first year of adoption of DTG use in Uganda, the Makerere University Infectious Diseases Institute (IDI) published multiple cases of mainly ART exposed people with HIV(PWH), presenting with accelerated hyperglycemia within weeks to a few months of starting DTG [5]. Since then, multiple other case reports of patients on INSTIs have been published as summarized in Table 1 [6–8]. Common to all cases, clinicians had to withhold the INSTI with eventual improvement in blood glucose. Following these case reports, the Uganda Ministry of Health guidelines recommended DTG substitution in all patients (both ART naïve and exposed) with incident T2DM [9], [10]. This blanket recommendation may have negative implications in programmatic HIV care especially in sub-Saharan Africa where the resistance to alternative anchor drugs like NNRTIs is > 10% in various countries, above the recommended WHO threshold [11], [12]. Whether DTG should be withheld in all PWH with incident T2DM is still unclear as is the mechanism responsible for accelerated hyperglycemia is still unclear. To highlight the shortcomings of this blanket recommendation to withhold DTG in these patients like in the Ugandan setting, we describe changes in blood glucose, glycated hemoglobin, insulin resistance and pancreatic beta cell function before and after withholding DTG in a patient who was diagnosed with T2DM at 36 weeks on DTG.

**Table 1:** Summary of published case reports of accelerated severe hyperglycemia in people with HIV on integrase inhibitors

Author (Country of origin of patients)	Baseline patient demographic and clinical characteristics	Clinical presentation	INSTI withheld?
Nathanial et al [15]. (North America)	25 y/male, ART experienced, switched to BIC/TAF/FTC -HBA1C-6.6%, FBG-85 mg/dl	After 4 months -DKA -HBA1C- >17% -Normal C-peptide	Yes -Marked improvement in glycemic control after withdrawal
	56y/male, ART experienced, switched to BIC/TAF/FTC -HBA1C-6%	After 2months: -DKA -HBA1C-12.6% -Normal C-peptide -preceding weight loss of 15 kg	Yes -Marked improvement in glycemic control after withdrawal
	41y/male, ART experienced, switched to BIC/TAF/FTC -FBG- 78 mg/dl -HBA1C- 5.6%	After 2 months: -DKA -Normal C-peptide -HBA1C- 12.4%	Yes -Marked improvement in glycemic control after withdrawal
Lamorde et al [5]. (Uganda)	-16 patients, 15 ART experienced, 1 ART naïve -Initiated on DTG anchored ART -Baseline blood glucose, HBA1C not measured	Mean duration before presentation to the ER: 4 months -DKA -preceding weight loss	Yes -Marked improvement in glycemic control after withdrawal
Fong et al [16] (North America)	-44y/male ART experienced -Switched to RAL -HBA1C- 2 months prior to switch – 5.5%	After 4 months: -DKA -HBA1C-9.3% -Normal C-peptide -preceding weight loss	Yes -Marked improvement in glycemic control after withdrawal
McLaughlin et al [8]. (North America)	-ART experienced -Switched to DTG -Known to have stage 4 CKD, Type II Diabetes Mellitus, Hypertension -HBA1C- 6.2%, FBG- 65 mg/dl	After 3 weeks: -Hyperosmolar Hyperglycemic state, RBG-949 mg/dl, HBA1C-14.9%.	Yes -Marked improvement in glycemic control after withdrawal.
Kamal et al [6]. (North America)	-ART experienced, defaulted in 2013. -started on TDF/FTC/ DTG in June- 2018 -HBA1C-5.9%	After 1 month: -On routine evaluation- RBG = 467 mg/dl (started on metformin 500 mg) After 2nd month: -Admitted with Hyperosmolar Hyperglycemic state, HBA1C-12.9%, started on insulin.	Not mentioned.

y- years ART- Anti-retroviral Therapy, BIC-bictegravir, TAF- tenofovir alafenamide fumarate, FTC-Lamivudine, DTG- dolutegravir. FBG-fasting blood glucose, HBA1C-glycated hemoglobin, DKA-diabetic ketoacidosis, RAL-raltegravir, RBG-random blood glucose, CKD-chronic kidney disease

## Case presentation

This 44-year-old man was enrolled in a prospective cohort study (**GL**ucose **Me**tabolism changes in Ugandan HIV patients on **D**olutegravir, **GLUMED** study) evaluating glucose metabolism changes in anti-retroviral therapy (ART) naïve PWH on tenofovir/ lamivudine/ DTG (TDF/3TC/ DTG) for 48 weeks [13]. PWH with normal 2-hour oral glucose tolerance tests (OGTTs) were enrolled for 48-week follow up with serial OGTTs at 12 and 36 weeks. The primary aim of the study was to determine incidence of T2DM in Ugandan PWH on DTG and describe glucose metabolism changes in patients with incident hyperglycemia versus those without.

### Baseline evaluation

At enrollment on ART, he reported no other known chronic illnesses and long-term medication. He was not severely immunocompromised with a baseline CD4 + cell count of 261cells/mm^3^ and was in WHO clinical stage I. Baseline viral load was not done. He had a normal baseline OGTT with fasting blood glucose (FBG)- 88.2 mg/dl and 2-hour blood glucose (2hBG) of 153 mg/dl and normal blood pressure. He did not have conventional risk factors for diabetes like family history of T2DM, obesity (body mass index (BMI) of 17.5 kg/m^2)^), anti- glutamic acid decarboxylase (anti-GAD) antibodies and insulin resistance (HOMA IR − 0.6 (normal < 2). He was a builder with calculated WHO metabolic equivalent minutes of 4800 per week on average (met the WHO recommended physical exercise recommendations for good physical health) [14]. However, he was positive for anti- Islet cell antigen 2 (anti-IA2) antibody (which is more predictive of type 1 DM) and had reduced pancreatic beta cell function (HOMA% β- 68% (normal = 100%)). Insulin resistance and pancreatic beta cell function was calculated using homeostatic modelling, a factor of serum fasting glucose and fasting insulin.

### Diagnosis of T2DM

During the 36-week study visit, he was diagnosed with T2DM basing on a 2hBG of 259 mg/dl. His BMI had increased to 20.9. Other tests included: HBA1C- 4.2%, HOMA IR- 2, HOMA% β- 101.4%. DTG was substituted with efavirenz according to the Uganda HIV treatment guidelines that recommended mandatory substitution of DTG in case of incident T2DM [9].

### Follow up after T2DM diagnosis and withholding of DTG

**Fig. 1:**
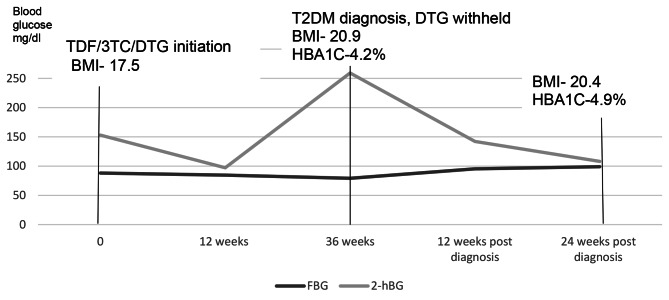
Changes in fasting and 2-hour blood glucose before and after withholding dolutegravir TDF/3TC/DTG- tenofovir/ lamivudine/ dolutegravir, BMI- body Mass Index, T2DM-Type 2 Diabetes Mellitus, HBA1C- glycated hemoglobin

**Fig. 2:**
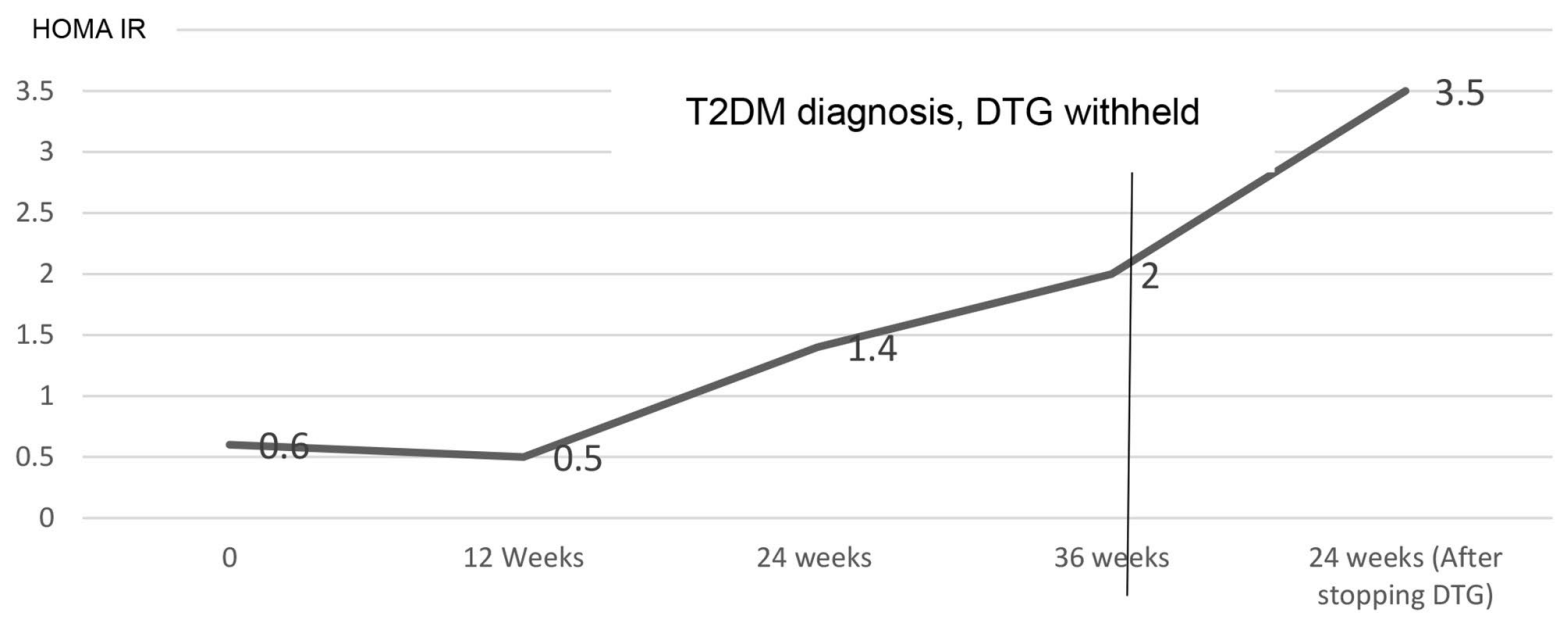
Changes in insulin resistance calculated by Homeostatic modelling (HOMA IR) before and after withholding dolutegravir HOMA IR- homeostatic model of insulin resistance, DTG- dolutegravir, T2DM-Type 2 Diabetes Mellitus

**Fig. 3:**
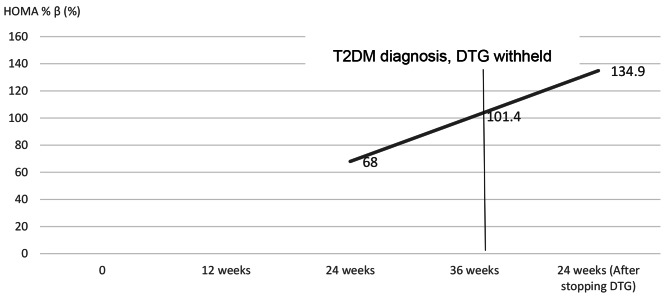
Changes in pancreatic beta cell function calculated by Homeostatic modelling (HOMA %β) before and after with-holding dolutegravir HOMA %β- Homeostatic model of pancreatic beta cell function, DTG- Dolutegravir, T2DM-Type 2 Diabetes Mellitus. (HOMA %β at baseline and 12 weeks was not calculated because the serum insulin was less than the HOMA calculator threshold)

He was managed on a low carbohydrate diet without T2DM pharmacologic intervention. Serial blood glucose evaluations at 2,4, 8, 12 and 24-weeks post T2DM diagnosis were within normal ranges. At 24 weeks, his HBA1C had increased to 4.9%. Insulin resistance continued to worsen with a HOMA IR of 3.5% while his pancreatic beta cell function continued to increase with HOMA %β = 134.9%. Dietary non-pharmacological management was continued after 24 weeks. Changes in BMI, blood glucose, HBA1C, HOMA IR and HOMA % β have been summarized in Figs. 1, 2 and 3.

## Discussion and conclusions

### What is known

Review of current literature suggests that at population level, integrase inhibitors are associated with a reduced risk of incident diabetes mellitus compared to NNRTIs and PIs [3], [4]. There is also evidence to demonstrate insignificant differences in effects on insulin resistance compared to other HIV drug classes [3]. Additionally, there are published case reports documenting that a certain section of heavily ART experienced PWH, probably with a currently unclear predisposition develop accelerated hyperglycemia when exposed to integrase inhibitors [5–8], [16]. These patients typically present with diabetic ketoacidosis preceded by weight loss, a phenotype typical of insulin deficiency states, but with normal C-peptide levels as summarized in Table 1. In these patients, the temporality between introduction of INSTIs and development of hyperglycemia is easily demonstrable. Similarly, withholding INSTIs has been demonstrated to lead to markedly reduced T2DM pharmacological treatment requirements and complete resolution of hyperglycemia in some cases (Table 1).

### The case presentation versus the cases in table 1

The patient we present had an asymptomatic onset of glucose intolerance over 36 weeks (diagnosed on a routine study visit) and gained weight before diabetes diagnosis in contrast to the above group of patients. Various factors such as immune reconstitution, improved appetite as well as dolutegravir itself could have contributed to the weight gain [17], [18]. Over the same period, he experienced worsening insulin resistance and concurrent increase in pancreatic beta cell function which could have been compensatory. Much as the worsening insulin resistance could be attributed to the weight gain, on evaluation of glucose changes in the whole study cohort, there was significant improvement in blood glucose despite a significant increase in BMI over the 48 weeks of follow up [13]. This may suggest that this patient had an inherent risk of developing T2DM, independent of weight gain and exposure to DTG. Blood glucose after T2DM diagnosis may have improved because of the dietary modification as well as compensatory hyperinsulinemia. It can as well be argued that blood glucose may have improved because DTG was discontinued but this may be unlikely because of the continued worsening insulin resistance and probable compensatory hyperinsulinemia.

### Limitations

The patient did not monitor his blood glucose at home after T2DM diagnosis hence the glucose measurements we documented were the only ones taken on study visit days. This is a single case report hence conclusions drawn much as insightful may not be generalizable to a larger population. Despite that, the patient was meticulously followed up with pertinent clinical data at the different time points.

## Conclusion

In conclusion, this case report suggests that dolutegravir may not have to be universally withheld in ART naïve PWH who develop diabetes mellitus but the decision made on a case-by-case basis. More research is however needed to ascertain the pathogenesis of accelerated hyperglycemia in a section of PWH on integrase inhibitors to inform clinical decisions on which PWH need drug substitution, if any. This is programmatically pertinent especially in sub-Saharan Africa where DTG is first line therapy in most HIV treatment programs and there is wide spread primary resistance to alternative drug classes like NNRTIs [19].

## Data Availability

The anonymized datasets used and/or analyzed during the current study are available from the corresponding author on reasonable request.

## List of abbreviations

DTG: Dolutegravir
T2DM: Type 2 Diabetes Mellitus
ART: Anti-retroviral therapy
INSTIs: Integrase strand transfer inhibitors
NNRTIs: Non-nucleoside transcriptase inhibitors
PIs: Protease inhibitors
CD4: Cluster of differentiation 4
HOMA IR: Homeostatic model for insulin resistance
HOMA %β: Homeostatic model for pancreatic beta cell function
FBG: Fasting blood glucose
2hBG: 2-hour blood glucose
HBA1C: Glycated hemoglobin
